# Glucosylceramide flippases contribute to cellular glucosylceramide homeostasis

**DOI:** 10.1016/j.jlr.2024.100508

**Published:** 2024-01-26

**Authors:** Natsuki Kita, Asuka Hamamoto, Siddabasave Gowda B. Gowda, Hiroyuki Takatsu, Kazuhisa Nakayama, Makoto Arita, Shu-Ping Hui, Hye-Won Shin

**Affiliations:** 1 Graduate School of Pharmaceutical Sciences, Kyoto University, Kyoto, Japan; 2 Faculty of Health Sciences, Hokkaido University, Sapporo, Japan; 3 Graduate School of Global Food Resources, Hokkaido University, Sapporo, Japan; 4 Laboratory for Metabolomics, RIKEN Center of Integrative Medical Sciences, Yokohama, Japan

**Keywords:** flippase, glucosylceramide, lipid transport, P4-ATPase, sphingolipid, glycosphingolipid

## Abstract

Lipid transport is an essential cellular process with importance to human health, disease development, and therapeutic strategies. Type IV P-type ATPases (P4-ATPases) have been identified as membrane lipid flippases by utilizing nitrobenzoxadiazole (NBD)-labeled lipids as substrates. Among the 14 human type IV P-type ATPases, ATP10D was shown to flip NBD-glucosylceramide (GlcCer) across the plasma membrane. Here, we found that conversion of incorporated GlcCer (d18:1/12:0) to other sphingolipids is accelerated in cells exogenously expressing ATP10D but not its ATPase-deficient mutant. These findings suggest that 1) ATP10D flips unmodified GlcCer as well as NBD-GlcCer at the plasma membrane and 2) ATP10D can translocate extracellular GlcCer, which is subsequently converted to other metabolites. Notably, exogenous expression of ATP10D led to the reduction in cellular hexosylceramide levels. Moreover, the expression of GlcCer flippases, including ATP10D, also reduced cellular hexosylceramide levels in fibroblasts derived from patients with Gaucher disease, which is a lysosomal storage disorder with excess GlcCer accumulation. Our study highlights the contribution of ATP10D to the regulation of cellular GlcCer levels and maintaining lipid homeostasis.

Membrane lipid flippases, which are members of the type IV P-type ATPase (P4-ATPase) family, establish and regulate the lipid asymmetry of cellular membranes and facilitate the translocation of lipids from extracellular pools. These enzymes are integral membrane proteins that utilize energy from ATP hydrolysis to translocate membrane lipids from the extracellular/luminal leaflet to the cytoplasmic leaflet of cellular membranes. There are 14 distinct P4-ATPases in humans with important differences in their tissue-specific expression, subcellular localization, and substrate specificity (1, 2, 3, 4). These differences in the localization and substrate specificity of P4-ATPases are important for coordinating physiological processes. Therefore, their impairment results in diverse pathologies (5). For example, ATP8B1 translocates phosphatidylcholine (PC) at the canalicular membrane in hepatocytes (6), and mutations in the *ATP8B1* gene lead to membrane damage and hepatic cholestasis (7, 8). ATP8A2 translocates phosphatidylserine (PS) in the central nervous system, and a mutation in the *ATP8A2* gene causes cerebellar ataxia mental retardation and disequilibrium syndrome in humans (9, 10).

P4-ATPases appear to translocate not only phospholipids but also glycosphingolipids. Sphingolipids consist of a long-chain sphingoid base (a sphingosine backbone) and ceramides are the basic units of all sphingolipids, such as SM and glycosphingolipids. Glycosphingolipids have carbohydrates attached to ceramides and glucosylceramide (GlcCer) is a major precursor of more complex glycosphingolipids, such as Gb3 and GM3 (11, 12, 13, 14, 15) (Supplemental Fig. S1). In addition to glycosphingolipids, GlcCer can be also transported to the plasma membrane (16). GlcCer is not only precursor lipid for complex glycosphingolipids but also bioactive lipid that can affect energy homeostasis and inflammation (11, 15, 17, 18, 19, 20, 21). ATP10D specifically translocates GlcCer, but not GalCer, at the plasma membrane (22), and ATP10B translocates PC and GlcCer in lysosomes (23). ATP10D is expressed ubiquitously, while ATP10B in limited tissues, such as the brain and gastrointestinal tract, as indicated by data from the Human Protein Atlas (24). Genome-wide association studies of European and Japanese populations have revealed strong associations between commonly found *ATP10D* single-nucleotide polymorphisms and the development of myocardial infarction and atherosclerosis, respectively (25, 26). Analysis of genetic screening data from patients with Parkinson’s disease has identified mutations in *ATP10B*, and these mutants are catalytically inactive (23). Therefore, the translocation of GlcCer between leaflets of the appropriate membranes is crucial for maintaining homeostasis in healthy individuals. However, it remains unclear how GlcCer translocation at the plasma membrane by ATP10D is associated with cellular functions and lipid homeostasis.

Gaucher disease (GD) is a lysosomal storage disorder with accumulation of GlcCer due to mutations in the lysosomal glucocerebrosidase (*GBA1*) gene (27, 28). The consequences of the deficiency are generally attributed to the accumulation of the substrate GlcCer, particularly in macrophages, and cells in the bone marrow, liver, and spleen (27). Primary treatment approaches for GD are supplying glucocerebrosidase to cells lack it and reducing excess GlcCer by employing GlcCer synthase inhibitors (27, 29).

Here, we demonstrate that the flippase activity of ATP10D is capable of translocating unmodified GlcCer (d18:1/12:0), which is then metabolized to generate other glycosphingolipid species. Additionally, we found that the expression of GlcCer flippases decreased the cellular hexosylceramide (HexCer) levels in cultured cells as well as in fibroblasts derived from patients with GD.

## Materials and methods

### Plasmids, antibodies, and reagents

Expression vectors for the C terminally hemagglutinin (HA)-tagged human ATP10D and ATP10D(E215Q) were previously described (22). A monoclonal rabbit anti-ATP1A1 antibody (EP1845Y) was purchased from Abcam (Cambridge). A monoclonal mouse anti-β-actin antibody (C4) was purchased from Santa Cruz (Dallas, TX); a monoclonal rat anti-HA antibody (3F10) was purchased from Roche Applied Science. AlexaFluor-conjugated secondary antibodies were purchased from Invitrogen. Cy3- and horseradish peroxidase–conjugated secondary antibodies were purchased from Jackson ImmunoResearch Laboratories (West Grove, PA). C12-GlcCer (C12 glucosyl(β) ceramide (d18:1/12:0)), nitrobenzoxadiazole (NBD)-GlcCer (N-{6-[(7-nitro-2-1,3-benzoxadiazol-4-yl)amino]hexanoyl}-D-erythro-sphingosine), NBD-PS (1-oleoyl-2-{6-[(7-nitro-2-1,3-benzoxadiazol-4-yl)amino]hexanoyl}-sn-glycero-3-phosphoserine), and the EquiSPLASH Lipidomix quantitative standard for mass spectrometry (containing ceramide (d18:1/15:0-d7) (internal standard)) were obtained from Avanti Polar Lipids (Ablabaster, AL). Solvents including methanol, isopropanol, and chloroform, of LC-MS grade were purchased from Wako Pure Chemical Industries, Ltd., (Osaka, Japan). Ammonium acetate solution (1 M) for LC-MS was purchased from Sigma-Aldrich (St. Louis, MO).

### Cell culture and establishment of stable cell lines

Fibroblasts from patients with type I GD (GM10915, homozygous L444P; designated GD-I), and a healthy individual (GM00498) were obtained from the Coriell Institute for Medical Research (Camden, NJ). These fibroblasts and HeLa cells were cultured in minimum essential medium (Nacalai Tesque, Kyoto, Japan) supplemented with 10% fetal calf serum (Gibco, Waltham, MA). Culture medium for fibroblasts was supplemented with minimum essential medium nonessential amino acids (Nacalai Tesque). HAP1, which is a near-haploid human cell line derived from KBM7, a human myeloid leukemia cell line, was obtained from the Haplogen and the cells were cultured in Isocove’s modified Dulbecco’s medium (Nacalai Tesque) supplemented with 10% fetal calf serum (Gibco). Fibroblasts with stable expression of C terminally HA-tagged ATP10D, ATP10B, and B-ATP10D, as well as amino acid–substituted ATP10D, ATP10B, and B-ATP10D were established according to previously described methods (6, 22). Immunoblot analysis was conducted as previously described (30, 31).

### Quantitative RT-PCR

Total RNA was isolated from HeLa, MDA-MB-231, HEK293T, HCT116, HepG2, MCF-7, Huh-7, HUVEC, BON, HAP1, KBM7, and human healthy fibroblasts (GM00498) using Isogen (TOYOBO) or RNeasy Mini Kit (Qiagen) and then subjected to reverse transcription using ReverTra Ace qPCR RT Master Mix (TOYOBO). The resultant cDNA was used as a template for PCR using THUNDERBIRD SYBR qPCR Mix (TOYOBO); fold changes in gene expression were normalized to the β-actin mRNA level. ATP10D and β-actin cDNAs were amplified using the following primer pairs: sense, 5′-catgaggttggagaacaaacttac-3′/antisense, 5′-ggggacaggaggctgaagta-3′; sense, 5′-gcaagagaggcatcctcacc-3′/antisense, 5′-cgtagatgggcacagtgtgg-3′, respectively.

### Immunofluorescence analysis

Cells were fixed with 3% paraformaldehyde in PBS for 30 min and immunostained as previously described (32) and visualized using an Axio Observer Z1 microscope (Carl Zeiss, Thornwood, NY).

### Cell-based flippase assay

Uptake of NBD-lipids was analyzed by flow cytometry as previously described (22). In brief, fibroblasts in a 6-well plate were washed and equilibrated at 15°C for 15 min in 1 ml of Hank’s balanced salt solution (pH 7.4) containing 1 g/L glucose (HBSS-glucose). The buffer was replaced with HBSS-glucose containing 600 μl of 1 μM NBD-lipid and cells were further incubated at 15°C. After incubation for the indicated times, the buffer was replaced with 600 μl of ice-cold PBS(−) (Nacalai Tesque) containing 2.5% (w/v) fatty acid–free BSA (Wako), 5 mM EDTA, and 0.5 μg/ml propidium iodide (Nacalai Tesque), and cells were incubated on ice for 30 min. Detached cells (>1 × 10^4^ cells/sample) were analyzed with a BD Accuri C6 Plus instrument (BD Biosciences) to measure the fluorescence of the incorporated and translocated NBD-lipids into the cytoplasmic leaflet of the plasma membrane.

### Incorporation of GlcCer (d18:1/12:0)

Cells were washed with HBSS-glucose (15°C) twice and incubated for 15 min at 15°C in HBSS-glucose. The initial concentration of C12-GlcCer in DMSO was 16 mM, which was diluted in HBSS-glucose to final concentration of 30 μM. Cells were incubated with 30 μM of C12-GlcCer in HBSS-glucose for 60 min at 15°C to prevent endocytosis and washed with ice-cold PBS++ (with 0.1 mM MgCl_2_ and 0.1 mM CaCl_2_). Cells were further incubated with 2.5% fatty acid–free BSA (Wako) in ice-cold PBS++ for 30 min on ice and washed with ice-cold PBS++ twice. The buffer was replaced with complete culture medium, and cells were incubated for the indicated times at 37°C. After incubation, cells were washed, harvested, and frozen until subsequent lipidome analysis.

### LC/MS analysis

A total of 5–6 ceramic beads (1.4 mm, Cat. No. 15-340-159, Fisherbrand, Pittsburgh, PA) and 100 μl methanol were added to a frozen cell pellet in a 1.5 ml Eppendorf tube and homogenized for 30 s × two cycles using a BeadMill 4 homogenizer (Fisherbrand). Total lipids were extracted from homogenates using the Folch method with modifications as previously established in our laboratory (33, 34). In brief, 100 μl internal standard (18.8 μM ceramide {d18:1/15:0 [d7]}) in methanol was added to 100 μl methanolic homogenate and vortexed at 3,500 rpm for 30 s. Thereafter, 400 μl chloroform and 100 μl Milli-Q were added and vortexed for 5 min. Extracts were centrifuged at 15,000 rpm for 10 min at 4°C and the organic layer was transferred to a new Eppendorf tube. The aqueous layer was re-extracted with additional 400 μl chloroform. The combined chloroform extracts were evaporated and 100 μl methanol was added to redissolve lipid residues. Approximately, 10 μl sample was injected into the LC-MS system.

Analysis was performed on a Prominence UFLC system (Shimadzu Corp, Kyoto, Japan) coupled with an LTQ Orbitrap mass spectrometer (Thermo Fisher Scientific Inc., San Jose, CA). Chromatographic separation was achieved using a reversed-phase Atlantis T3 C18 column (2.1 × 150 mm, 3 μm; Waters, Milford, MA) at an oven temperature of 40°C. The mobile phases were (A) Milli-Q containing 10 mM CH_3_COONH_4_, (B) isopropanol, and (C) methanol. A linear gradient was used as follows: 0–1 min, 30% B and 35% C; 1–14 min, 80% B and 10% C; 14–27 min, 85% B and 10% C; and 27–28 min, 30% B and 35% C. High-resolution mass spectrometry data were acquired in Fourier transform mode with a resolving power of 60,000, collision energy of 35 eV, and a scan range of 160–1900 *m*/*z*. Data-dependent acquisitions with tandem mass spectrometry were performed in an ion-trap mode at a precursor ion isolation width of 3 *m*/*z* and a collision energy of 40 eV. Quantification was performed after integrating the peaks using Xcalibur 2.2 software (Thermo Fisher Scientific Inc.), according to the guidelines of the Lipidomics Standards Initiative level 3 (https://lipidomics-standards-initiative.org/). Relative levels after normalizing the peak area by the protein content were calculated by determining the peak area ratios of the analytes to the internal standard and multiplying the peak area ratios using the amount of added internal standard.

### Gene editing using the CRISPR/Cas9 system

*ATP10D* or *CDC50A* gene in HAP1 cells was disrupted using the CRISPR/Cas9 system. Complementary oligonucleotides (for *ATP10D*, guide RNA (gRNA)-1, 5′-caccgctgatcagaggtgcaacca-3′ and 5′-aaactggttgcacctctgatcagc-3′ or gRNA-2 5′-caccgagagggagtctgctcacaa-3′, and 5′-aaacttgtgagcagactccctctc-3′; for *CDC50A*, 5′-caccgcggtgccccccggagcaca-3′ and 5′-aaactgtgctccggggggcaccgc-3′; target sequences are underlined) were synthesized and introduced into a *Bbs*I-digested vector PX459 (Addgene #48139) and the plasmids were transfected. The transfected cells were selected and cultured in a medium containing puromycin, and cell clones were isolated using SH800S cell sorter (SONY biotechnology). Genomic DNA was extracted from individual clones and subjected to PCR to amplify the region of interest using following primer sets (for ATP10D gRNA-1, 5′-atttcctgacatgcctggca-3′ and 5′-aggctccggagaacttctca-3′ (290 bp), and for gRNA-2, 5′-ttttccatggcggtcgtaaa-3′ and 5′-gtccgagaccgtaaagtgca-3′ (609 bp); for CDC50A, 5′-gggtggtccacctgcaaatcgc-3′, and 5′-cctcccctcacctcgatctcg-3′ (237 bp)). The KO was confirmed by direct sequencing of the amplified PCR product. Among the KO cells, some were diploid, and others were haploid because HAP1 cells are almost haploid cells (Supplemental Fig. S5).

## Results

### Conversion of incorporated GlcCer (d18:1/12:0) to other sphingolipids is accelerated in cells expressing ATP10D

**Fig. 1:**
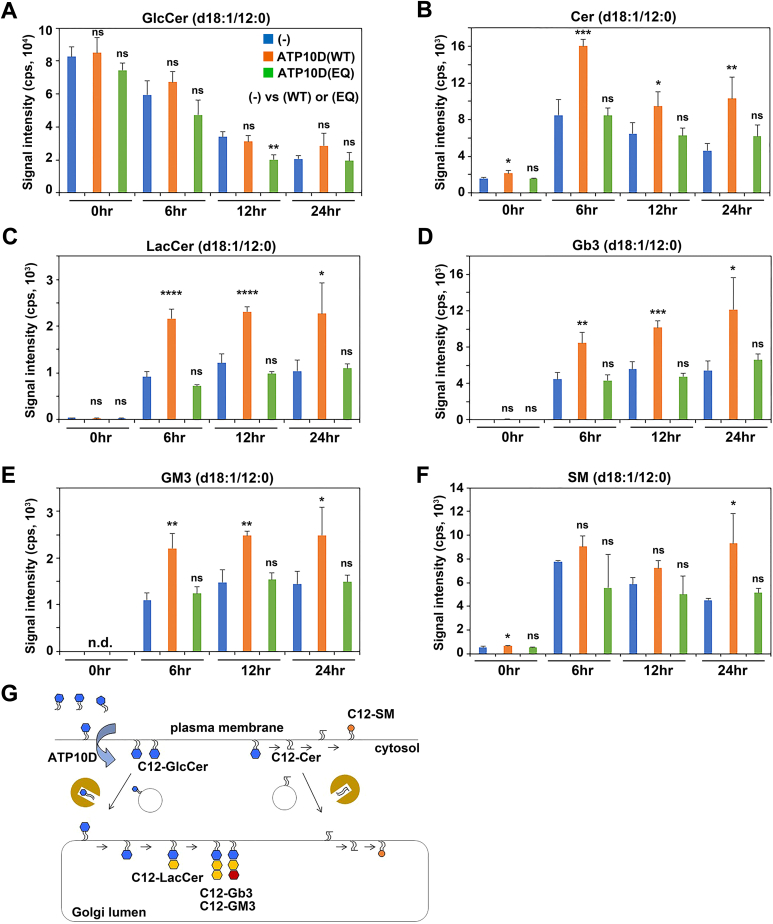
Turnover of C12-GlcCer in ATP10D-expressing HeLa cells. HeLa cells stably expressing C terminally HA-tagged ATP10D or the ATP10D(E215Q) mutant were incubated with C12-GlcCer (GlcCer{d18:1/12:0}) at 15°C for 60 min, followed by washing and further incubation in complete medium at 37°C for the indicated time periods. Total lipids were extracted from the individual cell pools, and a lipidomic analysis was performed to assess the turnover of the incorporated C12-GlcCer. A–F: Levels of GlcCer (A) and its metabolites (B–F) at the indicated time points. n.d., not detected. G: Schematic illustration of the turnover for the incorporated C12-GlcCer. Data are presented as mean ± SD of experiments performed in triplicate, except for SM (d18:1/12:0) in control cells (−) (which was performed in duplicate). One-way ANOVA was performed to assess variance and Dunnett analysis was performed for comparisons with the control (−). ∗*P* < 0.05, ∗∗*P* < 0.01, ∗∗∗*P* < 0.001, and ∗∗∗∗*P* < 0.0001. GlcCer, glucosylceramide; ns, not significant.

We have previously demonstrated that HeLa cells stably expressing ATP10D exhibit enhanced flipping activity toward NBD-labeled GlcCer (22). As, in the cell-based flippase assay, the NBD-labeled lipids located in the outer leaflet of the plasma membrane can be readily removed by treating the cells with fatty acid–free BSA, the levels of intracellular NBD-lipids resulting from their translocation from the outer to the inner leaflet can be measured using flow cytometry. However, there has been an ongoing debate because the behavior of NBD lipids may differ from that of native lipids due to the conjugation of NBD to the fatty acyl chain (35, 36). To investigate the ATP10D flippase activity toward unmodified GlcCer, we utilized a GlcCer with a medium fatty acyl chain (d18:1/12:0, designated as C12-GlcCer), which is rarely present in nature. HeLa cells stably expressing C terminally HA-tagged ATP10D or its ATPase-deficient mutant, ATP10D(E215Q), were treated with C12-GlcCer and its metabolites were determined through lipidomic analysis. The cells were incubated with C12-GlcCer at 15°C for 1 h, allowing C12-GlcCer to be incorporated into the plasma membrane. The incubation at 15°C prevents endocytosis. Subsequently, the cells were washed to remove the unincorporated C12-GlcCer and further incubated at 37°C for the indicated times (Fig. 1). The total cellular lipids were then extracted and subjected to lipidomic analysis. The cellular levels of C12-GlcCer were not significantly different between control HeLa cells and cells stably expressing ATP10D or ATP10D(E215Q) at 0 h, indicating the comparable incorporation of C12-GlcCer into the individual cell pools (Fig. 1A); the C12-GlcCer levels in the inner and outer leaflets could not be distinguished in these experiments. The incorporated C12-GlcCer levels gradually decreased as shown at the indicated time points (Fig. 1A).

Notably, after 6 h of incubation at 37°C, the levels of C12-ceramide (Cer) significantly increased in cells expressing ATP10D compared to control cells. However, no such increase was observed in cells expressing the ATP10D(E215Q) mutant (Fig. 1B). These results suggest that C12-GlcCer translocated to the cytoplasmic leaflet of the plasma membrane by the ATP10D flipping activity were directly converted into C12-Cer through the removal of the glucose moiety by cytoplasmic glucocerebrosidase (Fig. 1G and Supplemental Fig. S2) (37). Moreover, cells expressing ATP10D exhibited a notable increase in the levels of C12-lactosylceramide (LacCer), C12-Gb3, and C12-GM3 (Fig. 1C—E) compared to control cells and cells expressing the ATP10D(E215Q) mutant.

**Supplemental Figure S1:**
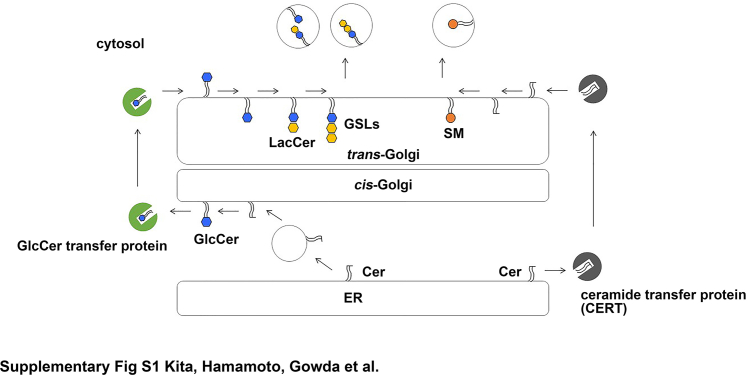
Illustration of turnover of sphingolipid biosynthesis in cells. Biosynthesis of sphingolipids. Ceramide synthesis occurs in the outer leaflet of the ER, and it is subsequently transported to the Golgi complex through two pathways: either via ceramide transfer protein (CERT) or vesicular transport. Ceramide transported via CERT, is converted to sphingomyelin by sphingomyelin synthase within the Golgi lumen. Ceramide transported via vesicular transport, is converted to glucosylceramide in the cytoplasmic leaflet of the Golgi and transferred to the *trans*-Golgi area by glucosylceramide transfer protein, such as GLTP and/or FAPP2. Subsequently, glucosylceramide is translocated (flopped) into the Golgi lumen by an unknown transporter and serve as a precursor for the synthesis of various glycosphingolipids. Synthesized sphingolipids (glycosphingolipids, sphingomyelin) at the Golgi complex are delivered to other cellular membranes by vesicular transport. Cer, ceramide; ER, endoplasmic reticulum; GlcCer, glucosylceramide; GSLs, glycosphingolipids; LacCer, lactosylceramide; SM, sphingomyelin.

**Supplemental Figure S2:**
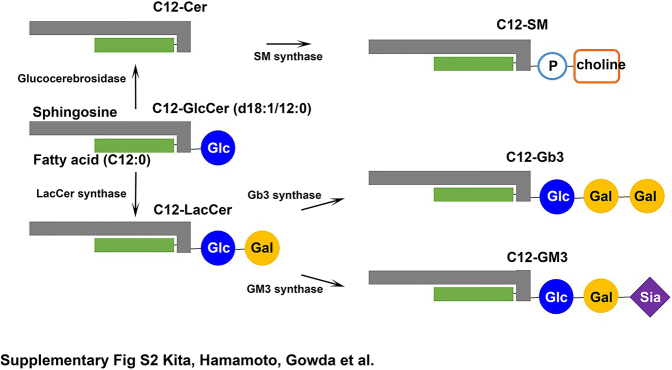
Illustration of C12-GlcCer(d18:1/12:0) turnover in cells. Cellular turnover of incorporated C12-GlcCer. Sphingosine backbone (gray), C12 fatty acyl chain (green). Cer, ceramide; Gal, galactose; Glc, glucose; GlcCer, glucosylceramide; LacCer, lactosylceramide; Sia, sialic acid; SM, sphingomyelin.

In the course of glycosphingolipid biosynthesis, GlcCer is synthesized at the cytoplasmic leaflet of the Golgi complex and subsequently translocated (flopped) into the luminal leaflet of the Golgi complex (Supplemental Fig. S1). Following the translocation, GlcCer undergoes conversion into LacCer, which is further metabolized to generate complex glycosphingolipids, such as Gb3 and GM3 (Supplemental Fig. S1) (11, 12, 13, 14, 15). C12-GlcCer, which was translocated to the cytoplasmic leaflet of the plasma membrane through the ATP10D activity, can be transported to the Golgi and flopped into the lumen (Fig. 1G). Subsequently, the C12-GlcCer can be converted into C12-LacCer through the addition of galactose in the Golgi complex lumen (Fig. 1G and Supplemental Fig. S2). The C12-LacCer can be further metabolized into C12-Gb3 and C12-GM3 in the Golgi lumen through the addition of galactose and N-acetyl neuraminic acid, respectively (Fig. 1G and Supplemental Fig. S1). Given that the levels of metabolites of C12-GlcCer, such as C12-Cer, C12-LacCer, C12-Gb3, and C12-GM3, increased in cells expressing ATP10D but not in those expressing its ATPase-deficient mutant, the translocated (flipped) C12-GlcCer in the inner leaflet of the plasma membrane, facilitated by ATP10D flippase activity, efficiently undergoes conversion into other metabolites (Fig. 1B–E, G). C12-Cer can be further converted into C12-SM in the luminal leaflet of the Golgi complex or at the outer leaflet of the plasma membrane through the addition of phosphocholine by SM synthases (Fig. 1G); however, this increase is only observed at a later time point (24 h) (Fig. 1F). Taken together, these results suggests that ATP10D can translocate extracellular GlcCer into the cytoplasmic leaflet and the flipped GlcCer is subsequently converted to other metabolites. Moreover, these results provide the first evidence that ATP10D is involved in the translocation of unmodified GlcCer as well as NBD-GlcCer (22) from the outer to the inner leaflet of the plasma membrane.

### Exogenous expression of ATP10D in HeLa cells decreases the cellular HexCer levels

**Fig. 2:**
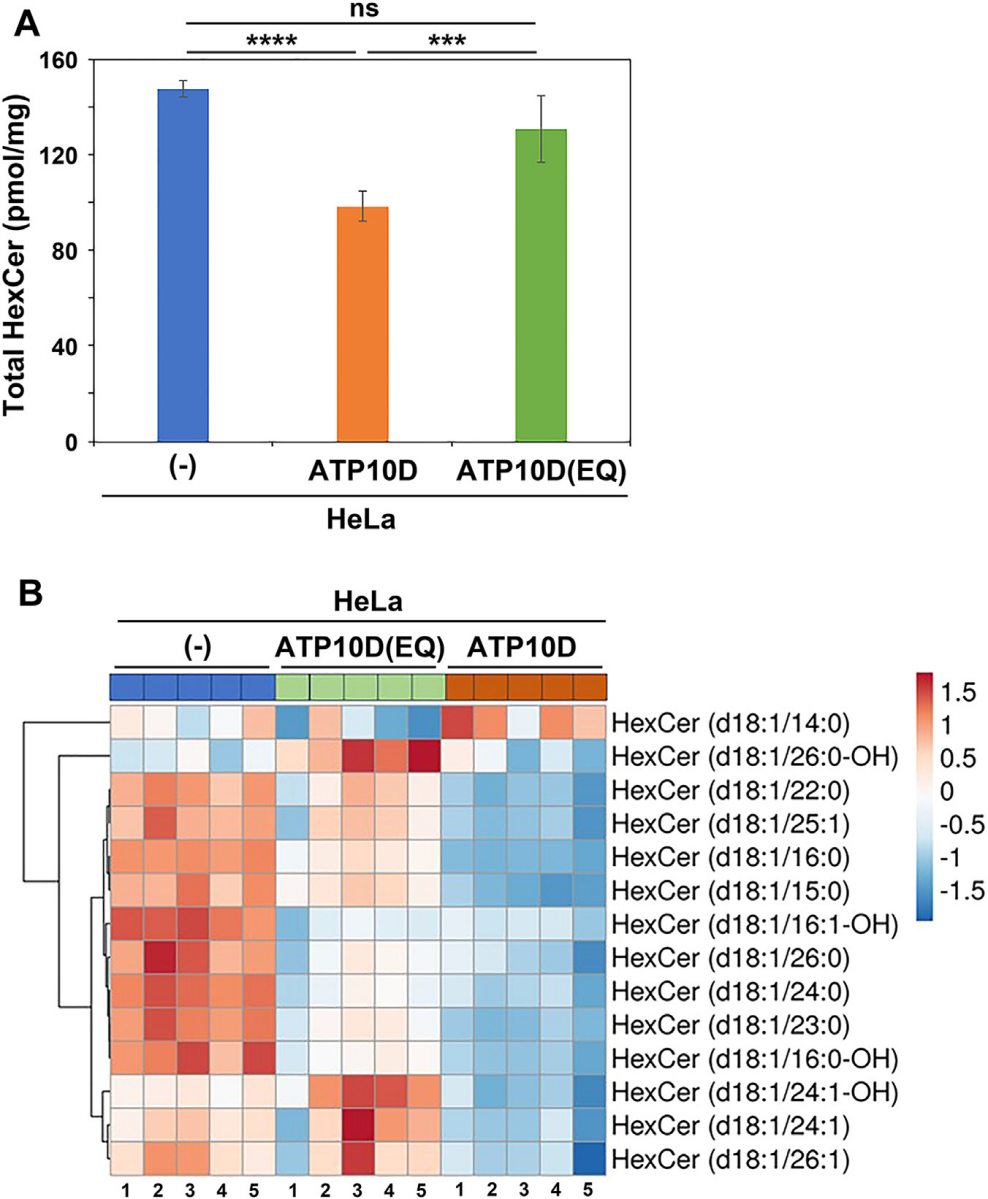
Cellular HexCer levels in ATP10D-expressing HeLa cells. A: Total lipids were extracted from control HeLa cells (−) and HeLa cells stably expressing ATP10D-HA and the ATP10D(E215Q)-HA mutant. Data are represented as mean ± SD (each sample was tested in quintuplicate). One-way ANOVA was performed to assess variance, and Tukey’s post hoc analysis was performed for comparisons between groups. ∗∗∗*P* < 0.001 and ∗∗∗∗*P* < 0.0001. ns, not significant. B: The ClustVis, a web tool, was used to generate the heatmap representing HexCer species in the individual cell pools. The numbers 1–5 correspond to the quintuplicated samples. HexCer, hexosylceramide.

**Supplemental Figure S3:**
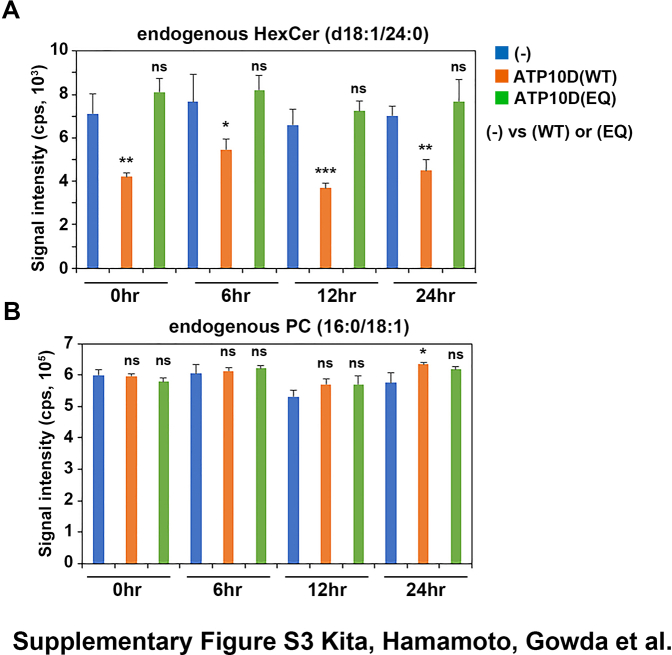
Endogenous HexCer and PC levels. Relative amount of the major endogenous HexCer species (d18:1/24:0) (**A**) and PC species (16:0/18:1) (**B**) are shown at the indicated time points (related to Fig. 1). Data are presented as mean ± SD of experiments performed in triplicate. One-way ANOVA was performed to assess variance and Dunnett analysis was performed for comparisons with the control (-). ∗*p* < 0.05, ∗∗*p* < 0.01, ∗∗∗*p* < 0.001. ns, not significant.

During the course of these experiments, we unexpectedly observed a significant decrease in the level of major endogenous HexCer species (d18:1/24:0) in cells expressing ATP10D, but not in those expressing the ATP10D(E215Q) mutant (Supplemental Fig. S3A). The level of major endogenous PC species (16:0/18:1) was not largely altered (Supplemental Fig. S3B). Although the lipidomic analysis did not allow us to distinguish between GlcCer and galactosylceramide, it is widely known that the majority of HexCer found in mammalian cells and tissues, except for the nervous system, is composed of GlcCer (38). Therefore, in this manuscript, we presumed HexCer in HeLa cells as GlcCer. It is important to note that the detected HexCer levels are relative rather than absolute. To validate the phenotype shown in Supplemental Fig. S3A, we examined the total HexCer levels in HeLa cells expressing ATP10D and ATP10D(E215Q) mutant under steady-state conditions (Fig. 2). The cellular HexCer levels were found to be significantly reduced in cells expressing ATP10D but no such decrease was observed in cells expressing ATP10D(E215Q) mutant (Fig. 2A). Additionally, most HexCer species exhibited a tendency to decrease in ATP10D expressing cells (Fig. 2B). These observations suggest the potential role of ATP10D activity in the regulation of cellular GlcCer levels.

### Knockout of *ATP10D* or *CDC50A* decreases endogenous GlcCer-flipping activity in HAP-1 cells

**Fig. 3:**
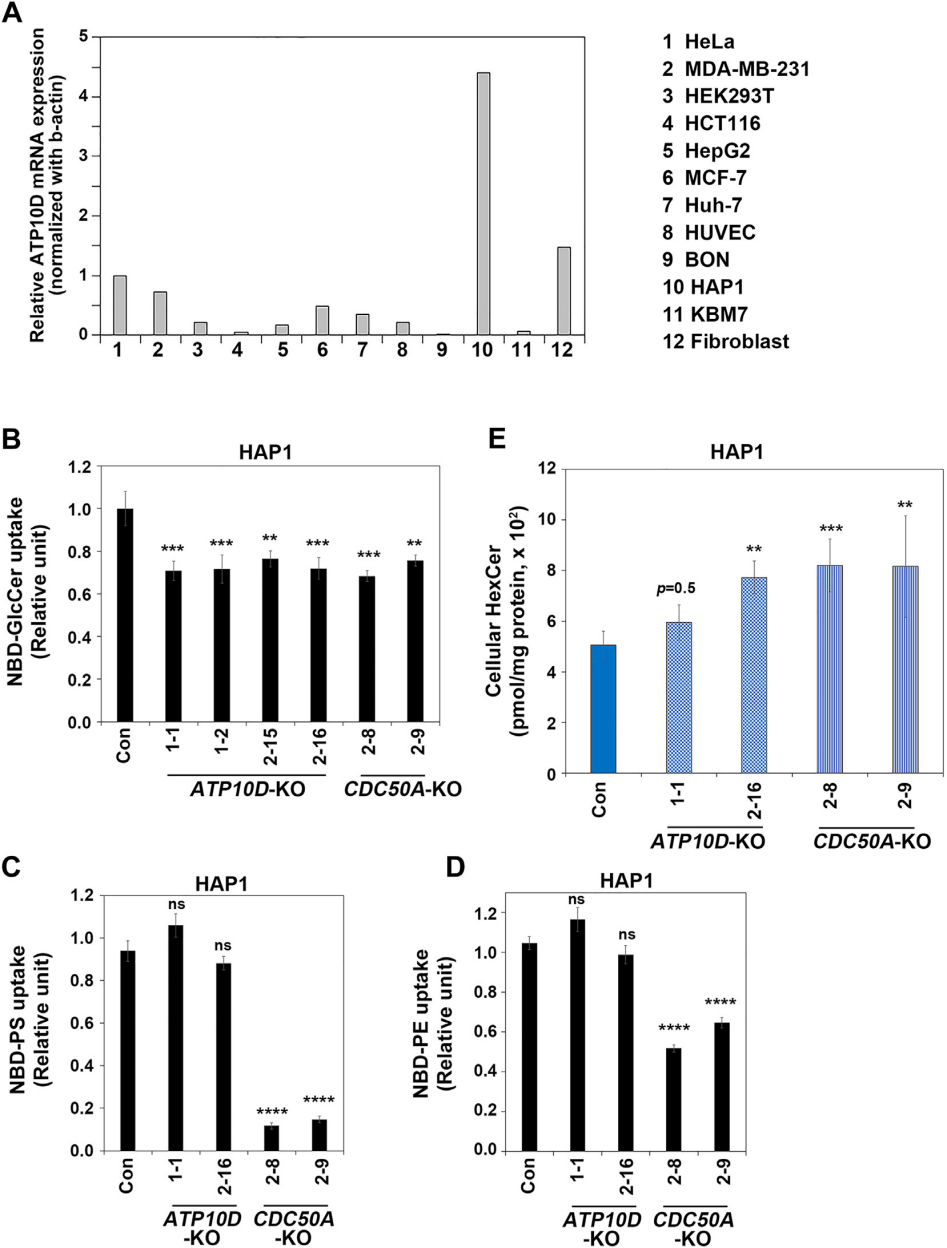
Cellular HexCer levels in *ATP10D*-KO and *CDC50A*-KO HAP1 cells. A: Quantitative real-time PCR analysis of *ATP10D* mRNA levels in various human cell lines. Total RNA was extracted from the indicated cell lines, and ATP10D mRNA expression levels were quantified using real-time PCR. The data are presented as the relative expression levels of *ATP10D* mRNA normalized to that of *β-actin*. The results shown are representative of two independent experiments, and each data represents the mean of experiments performed in triplicate. B–D: HAP1 (near haploid) cells knocked out of ATP10D or CDC50A were subjected to flippase assay. The cells were washed with flippase assay buffer and incubated with NBD-GlcCer for 30 min (B), NBD-PS for 5 min (C), or NBD-PE for 15 min (D) at 15°C. After extraction with fatty acid–free BSA, the residual fluorescence intensity associated with the cells was determined by flow cytometry. Graphs display the mean ± SD from three independent experiments. One-way ANOVA was performed to assess variance, and comparisons with parental HAP1 cells (Con) were performed using Tukey’s post hoc analysis. ∗∗*P* < 0.01, ∗∗∗*P* < 0.001, and ∗∗∗∗*P* < 0.0001; ns, not significant. E: Total lipids were extracted from parental HAP1 cells (Con) and the individual KO HAP1 cell lines. Data are presented as the mean ± SD (each sample was tested in quintuplicate). One-way ANOVA was performed to assess the variance, and comparisons with control HAP1 cells were performed using Tukey’s post hoc analysis. ∗∗*P* < 0.01 and ∗∗∗*P* < 0.001. GlcCer, glucosylceramide; HexCer, hexosylceramide; NBD, nitrobenzoxadiazole; PE, phosphatidylethanolamine; PS, phosphatidylserine.

**Supplemental Figure S4:**
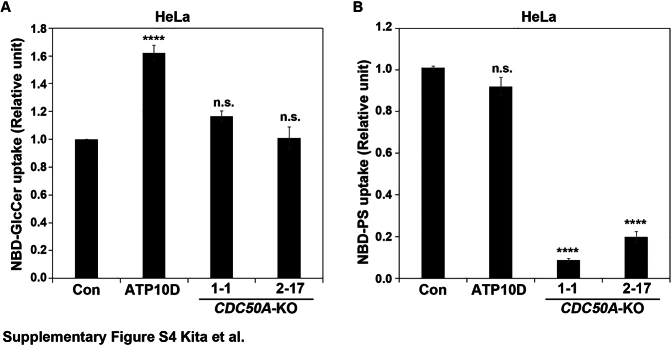
GlcCer-flippase activity in *CDC50A*-KO HeLa cells. HeLa cells stably expressing the ATP10D-HA, and HeLa cells knocked out of CDC50A were incubated with the indicated NBD-lipids at 15°C for 30 min (NBD-GlcCer) and 5 min (NBD-PS). After extraction with fatty acid-free BSA, the residual fluorescence intensity associated with the cells was measured by flow cytometry. The graph displays the average ± SD from three independent experiments. A one-way ANOVA was performed to assess the variance, and comparisons with parental HeLa cells (control) were performed using Tukey’s post-hoc analysis. ∗∗∗∗*p* < 0.0001, ns, not significant.

To further investigate the role of ATP10D in the regulation of the cellular GlcCer levels, we tried to examine the total HexCer levels in *CDC50A*-KO HeLa cells, which were established previously (39). CDC50A is necessary for the proper subcellular targeting of most P4-ATPases, including ATP10D (31). We firstly examined two different *CDC50A*-KO cell clones to detect the endogenous flipping activity toward NBD-GlcCer across the plasma membrane. However, the flipping activity toward NBD-GlcCer was not significantly decreased in *CDC50A*-KO cells compared to the parental HeLa cells (control), although the flipping activity toward NBD-PS was dramatically decreased (Supplemental Fig. S4). Therefore, the endogenous GlcCer-flippase activity was not detectable in HeLa cells. It is possible that the intrinsic GlcCer-flippase activity may not reach a detectable level at the steady state in HeLa cells. We speculated that the endogenous GlcCer-flippase activity might become detectable in cells expressing higher levels of ATP10D compared to HeLa cells. We quantitatively examined the ATP10D mRNA levels in the various cell lines using RT-PCR. ATP10D is known to be ubiquitously expressed in numerous cell types and tissues, with notably high expression levels detected in the placenta according to the Human Protein Atlas (24). Among the various cell lines examined, HAP1 cells exhibited considerably high expression levels of ATP10D (Fig. 3A). Therefore, we hypothesized that the endogenous GlcCer-flipping activity could be detectable in HAP1 cells.

**Supplemental Figure S5:**
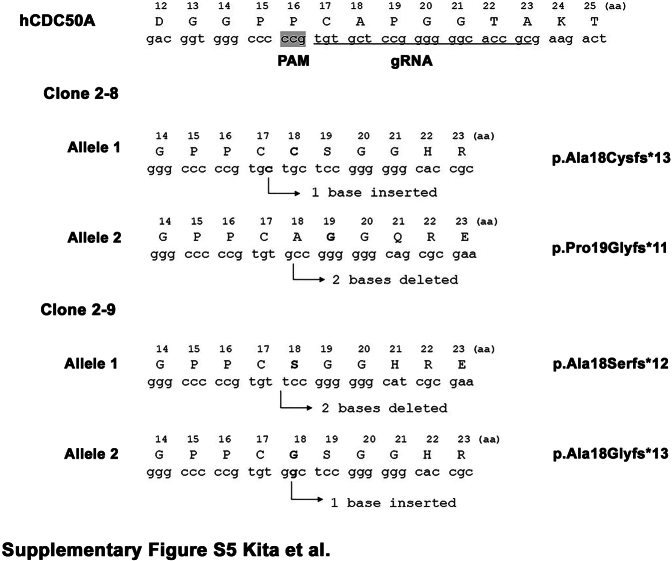
Disruption of *CDC50A* by CRISPR/Cas9 in HAP1 cells. To confirm the editing of *CDC50A* in HAP1 cells (near haploid), genomic DNA was extracted from individual clones and subjected to PCR. The KO was confirmed by direct sequencing of the amplified PCR products. Both clones carry biallelic indels indicating that some HAP1 cells have two alleles.

**Supplemental Figure S6:**
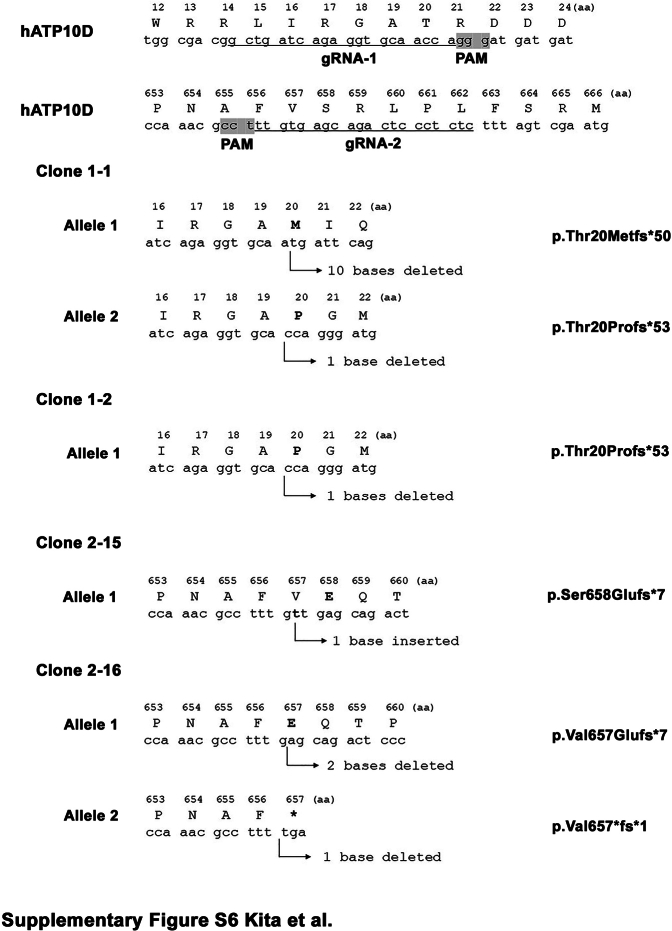
Disruption of *ATP10D* by CRISPR/Cas9 in HAP1 cells. To confirm the editing of *ATP10D* in HAP1 cells (near haploid), genomic DNA was extracted from individual clones and subjected to PCR. The KO was confirmed by direct sequencing of the amplified PCR products. Two clones carry biallelic indels and the other two clones carry monoallelic indels indicating that some HAP1 cells have two alleles.

We established *ATP10D*-KO and *CDC50A*-KO HAP1 cells using a CRISPR/Cas9 system. Successful KO was confirmed through genomic PCR and sequencing (Supplemental Figs. S5 and S6). We assessed the flipping activity toward NBD-GlcCer in four *ATP10D*-KO and two *CDC50A*-KO HAP1 cell clones and compared them to control cells (Fig. 3B). The flipping activity toward NBD-GlcCer was significantly decreased in all *ATP10D*-KO and *CDC50A*-KO cell clones (Fig. 3B). The reduction in the GlcCer-flippase activity was comparable between the *ATP10D*-KO and *CDC50A*-KO HAP1 cells, suggesting that ATP10D might be the primary GlcCer flippase within the P4-ATPase family in the plasma membrane. We also examined the flipping activities toward NBD-PS and NBD-phosphatidylethanolamine in *ATP10D*-KO and *CDC50A*-KO HAP1 cells (Fig. 3C and D). Uptake of NBD-PS or NBD-phosphatidylethanolamine decreased in *CDC50A*-KO HAP1 cells but was not significantly affected in *ATP10D*-KO cells (Fig. 3C, D), confirming substrate selectivity of ATP10D as previously described (22, 31).

### Knockout of *ATP10D* or *CDC50A* in HAP1 cells increases the cellular HexCer levels

To investigate the involvement of ATP10D in the regulation of cellular GlcCer levels, we performed lipidomic analysis using two KO clones of each gene. We observed a significant elevation in the total cellular HexCer levels in one *ATP10D*-KO clone (2, 3, 4, 5, 6, 7, 8, 9, 10, 11, 12, 13, 14, 15, 16) and both *CDC50A*-KO clones (2-8 and 2-9) compared to control cells (Fig. 3E). While the increase in the cellular HexCer levels was not statistically significant in another *ATP10D*-KO clone (1-1), a tendency toward elevated HexCer levels was observed. Taken together, these findings suggest that the increase in the GlcCer content at the cytoplasmic leaflet of the plasma membrane through the exogenous expression of ATP10D contributes to a decrease in the cellular HexCer levels, while a decrease in the GlcCer content at the cytoplasmic leaflet through the depletion of ATP10D is associated with an increase in the cellular HexCer levels.

### Expression of GlcCer flippases reduces the cellular HexCer levels in GD fibroblasts

We examined whether exogenous expression of ATP10D can also reduce the cellular HexCer levels in other cell types. To this end, we used fibroblasts derived from patients with GD. GD results from the impaired enzymatic activity of lysosomal glucocerebrosidase, which is encoded by the *GBA1* gene and is responsible for the removal of the glucose moiety from GlcCer (27, 28). Consequently, GlcCer and/or its derivatives accumulate in lysosomes of GD cells (40, 41).

**Fig. 4:**
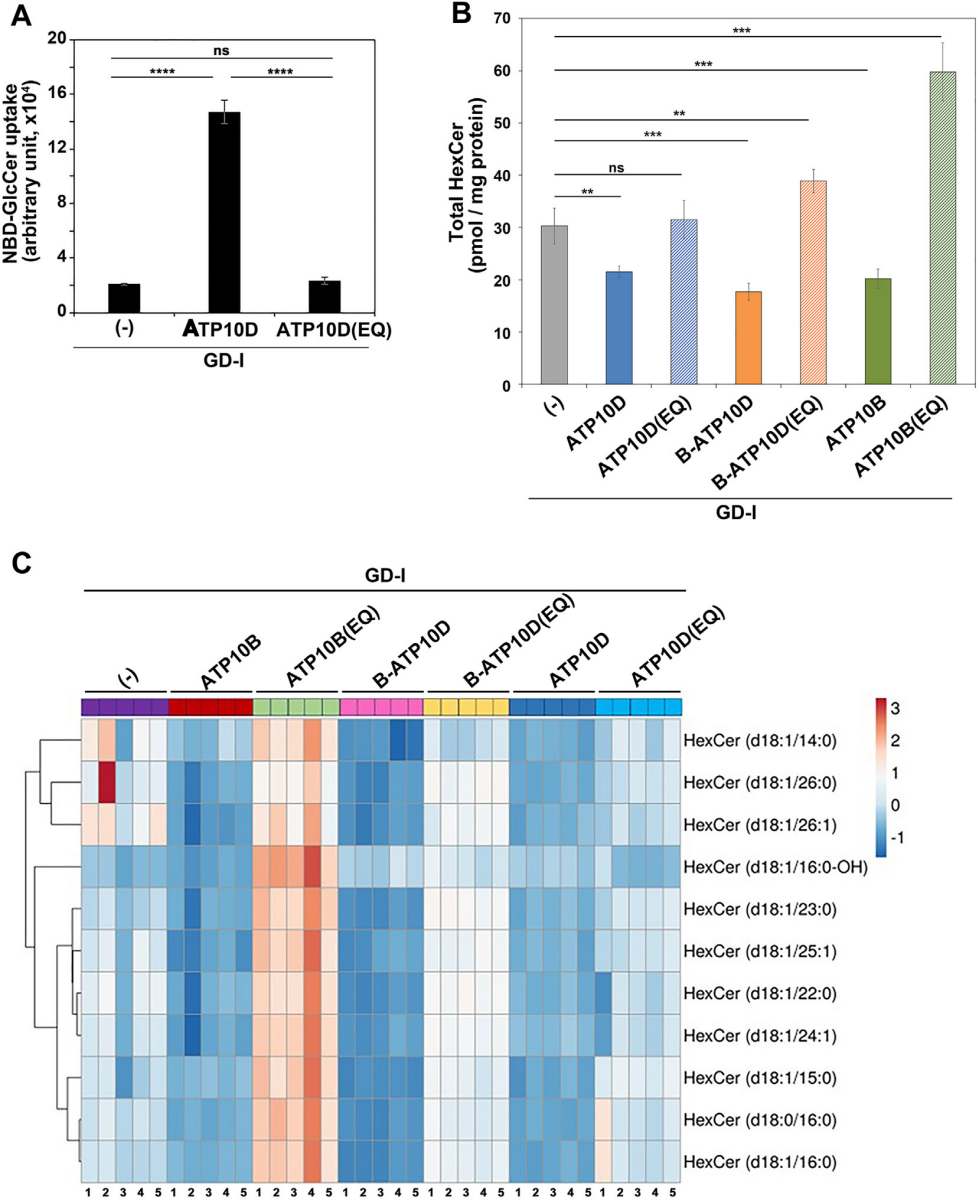
Cellular HexCer levels in GD-I cells expressing GlcCer flippases. A: GD-I fibroblasts stably expressing the C terminally HA-tagged ATP10D and ATP10D(E215Q) mutant were incubated with NBD-GlcCer for 30 min at 15°C. The residual fluorescence intensity associated with the cells was determined by flow cytometry after extraction with fatty acid–free BSA. Graphs display averages from three independent experiments ± SD. One-way ANOVA was performed to assess the variance, and comparisons were performed using Tukey’s post hoc analysis. ∗∗∗∗*P* < 0.0001. ns, not significant. B: Total lipids were extracted from the parental GD-I fibroblasts and the cells stably expressing the C terminally HA-tagged ATP10D, ATP10B, B-ATP10D, and their ATPase-deficient mutants, and lipidomic analysis was performed. Data are presented as the mean ± SD (each sample was tested in quintuplicate). One-way ANOVA was performed to assess the variance, and comparisons were performed using Tukey’s post hoc analysis. ∗∗*P* < 0.01 and ∗∗∗*P* < 0.001. ns, not significant. C: The ClustVis, a web tool, was used to generate the heatmap representing HexCer species in the individual fibroblast pools. The numbers 1–5 correspond to the quintuplicated samples. GlcCer, glucosylceramide; GD, Gaucher disease; HexCer, hexosylceramide; NBD, nitrobenzoxadiazole.

**Supplemental Figure S7:**
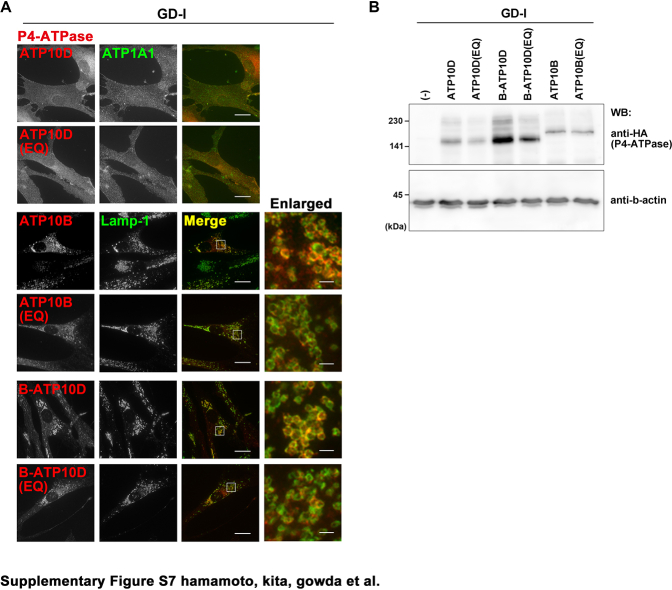
Expression and localization of ATP10s in type I GD fibroblasts. Fibroblasts derived from a patient with type I GD (GD-I) stably expressing the C-terminally HA-tagged ATP10D, ATP10B, B-ATP10D, and their E-to-Q mutants were analyzed. (**A**) Cells were fixed, permeabilized, and incubated with anti-HA and anti-ATP1A1 or anti-Lamp-1 antibodies followed by Cy3-conjugated anti-rat and Alexa Fluor 488-conjugated anti-rabbit or anti-mouse secondary antibodies. Bars, 20 μm and 2 μm in enlarged images. (**B**) Expression levels of each ATP10 protein in GD-I cells were analyzed by immunoblotting with anti-HA and anti-β-actin (as an internal control) antibodies.

When type I GD (GD-I) fibroblasts stably expressing C terminally HA-tagged ATP10D and ATP10D(E215Q) were established, an increase in the flipping activity toward NBD-GlcCer was confirmed in cells expressing ATP10D but not in those expressing ATP10D(E215Q) (Fig. 4A). The expression levels of ATP10D and its mutant, and their localization to the plasma membrane were confirmed by immunoblot and immunofluorescence analysis, respectively (Supplemental Fig. S7). Notably, the expression of ATP10D led to a decrease in the total cellular HexCer levels in GD-I fibroblasts (Fig. 4B, C), like in HeLa cells (Fig. 2A and Supplemental Fig. S3A). By contrast, the expression of ATP10D(E215Q) did not decrease the HexCer levels. These findings suggest that the enhancement of GlcCer flipping activity at the plasma membrane generally gives rise to a reduction in the cellular HexCer levels.

Since GlcCer could be largely accumulated in lysosomes of GD cells, we next examined whether the expression of lysosomal GlcCer flippases also reduces the cellular HexCer levels in GD-I fibroblasts. We previously demonstrated the localization of ATP10B to lysosomes (31). Moreover, we showed that a chimeric protein, B-ATP10D, in which the N-terminal cytoplasmic region of ATP10D is substituted by that of ATP10B, exhibits localization to lysosomes instead of the plasma membrane (42). Since ATP10D specifically flips NBD-GlcCer in the cell-based flippase assay (Fig. 4A) and ATP10B was shown to flip NBD-GlcCer and NBD-PC in microsomes (23), ATP10B and B-ATP10D are thought to translocate GlcCer from the luminal to the cytoplasmic leaflet in lysosomes. GD-I fibroblasts stably expressing the lysosomal GlcCer flippases ATP10B, B-ATP10D, and their E-to-Q mutants with an HA-tag were established. ATP10B, B-ATP10D, and their EQ mutants were observed to localize to Lamp-1-positive compartments in GD-I fibroblasts (Supplemental Fig. S7A). The expression levels of the individual ATP10 constructs were confirmed through immunoblot analysis (Supplemental Fig. S7B). Lipidomic analysis showed that the cellular HexCer levels were reduced by the exogenous expression of ATP10B or B-ATP10D (Fig. 4B, C). In contrast, the HexCer levels were not decreased by the expression of either of the EQ mutants; rather, the levels were elevated through the expression of these mutants compared to GD-I fibroblasts (Fig. 4B). These results demonstrate that increasing GlcCer-flipping activity in lysosomes as well as at the plasma membrane by the expression of P4-ATPases reduces the cellular HexCer levels in GD fibroblasts.

## Discussion

**Fig. 5:**
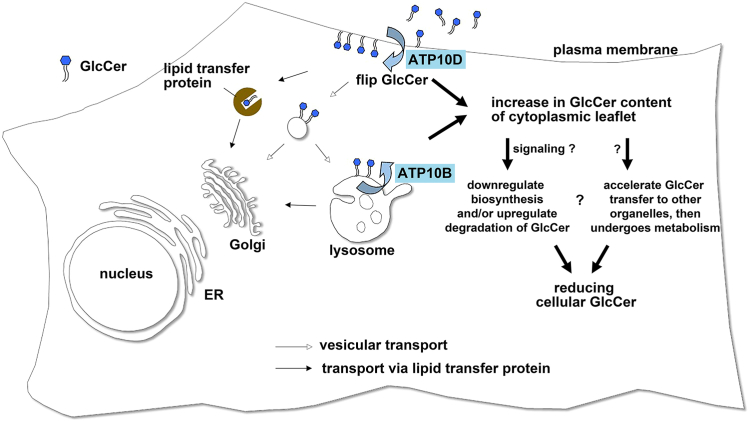
Schematic model of a decrease in the cellular GlcCer levels in cells expressing GlcCer flippases. Exogenous expression of GlcCer flippases, ATP10D or ATP10B, leads to an increase in the GlcCer levels in the cytoplasmic leaflet of the plasma membrane or lysosomes, respectively. The elevated cytoplasmic GlcCer may transduce signals to downregulate cellular GlcCer levels, thereby contributing to the maintenance of GlcCer homeostasis. Alternatively, GlcCer present in the cytoplasmic leaflet may be transferred to other organelles, such as the Golgi complex or the endoplasmic reticulum, facilitated by lipid-transfer proteins and/or vesicular transport. Once transferred, GlcCer can undergo further metabolism. GlcCer, glucosylceramide.

In this study, we made an important observation regarding the metabolism of C12-GlcCer that were incorporated into cells. Notably, we found that the conversion of incorporated C12-GlcCer to other metabolites was significantly accelerated in cells expressing ATP10D compared to those expressing its ATPase-deficient mutant. These findings suggest that the flippase activity of ATP10D facilitates the translocation of C12-GlcCer to the cytoplasmic leaflet of the plasma membrane, thereby efficiently enabling its subsequent conversion into other metabolites (Fig. 1G). This is the first experimental evidence demonstrating the ability of ATP10D to translocate intact GlcCer (C12-GlcCer), in addition to NBD-GlcCer, at the plasma membrane. Furthermore, our results demonstrate that the recognition of GlcCer by ATP10D is dependent on the headgroup, whereas the length of the fatty acyl chain may not be essential for the recognition. Considering that C12-GlcCer, which is translocated (flipped) to the cytoplasmic leaflet of the plasma membrane, can undergo conversion to other glycosphingolipids, the GlcCer flippase activity at the plasma membrane could influence the cellular GlcCer metabolism. Indeed, we found that the exogenous expression of ATP10D led to a decrease in the cellular HexCer levels, whereas the depletion of ATP10D resulted in elevated cellular HexCer levels. Although it remains unknown whether GlcCer distributes asymmetrically between the leaflets of the plasma membrane, an increase in the GlcCer content in the cytoplasmic leaflet of the plasma membrane, may correlate with a reduction in the cellular HexCer levels (Fig. 5). We presumed HexCer in HeLa cells and fibroblasts as GlcCer because the majority of HexCer found in mammalian cells and tissues, except for the nervous system, is composed of GlcCer (38, 43).

It is noteworthy that the expression of lysosomal GlcCer flippases but not their ATPase-deficient mutants, led to a reduction in the cellular HexCer levels in the GD fibroblasts. This finding suggests that the increased GlcCer content in the cytoplasmic leaflet, not only at the plasma membrane but also at lysosomes, contributes to a decrease in the cellular HexCer levels. Lysosomal GlcCer levels might remain at lower levels in healthy cells due to the presence of active GBA1 enzyme. By contrast, lysosomal GlcCer levels are elevated in GD cells. Consequently, the stable expression of lysosomal GlcCer flippases in GD cells could potentially lead to an increase in cytoplasmic GlcCer content in lysosomes. How does expression of GlcCer flippases decrease the cellular HexCer levels? One possibility is that the increased cytoplasmic GlcCer resulting from enhanced flipping activity triggers a signaling cascade that leads to a reduction in the cellular HexCer levels. This reduction may involve an increase in HexCer degradation and/or a decrease in HexCer synthesis, thereby contributing to cellular glycosphingolipid homeostasis (Fig. 5). The second possibility is that the elevated cytoplasmic GlcCer may accelerate its transfer to other organelles, including the Golgi complex and the endoplasmic reticulum, facilitated by lipid-transfer proteins, such as glycolipid transfer protein (GLTP) (44, 45), or via vesicular transport. GLTP interacts with VAP-A, which localizes to the endoplasmic reticulum and interacts with various lipid transfer (or exchange) proteins in membrane contact sites (46, 47, 48), and GLTP is also involved in membrane trafficking (49). Subsequently, the transferred GlcCer can be metabolized to generate other sphingolipid species (Fig. 5). Indeed, the incorporated C12-GlcCer was found to be converted into other glycosphingolipids, such as LacCer, Gb3, and GM3, which are produced in the Golgi lumen (Fig. 1G). However, it is still unknown the mechanism underlying the sensing of GlcCer levels in the cytoplasmic leaflet (Fig. 5). Given that ATP10D exhibits ubiquitous expression, while ATP10B is limited to specific tissues (24) and cell lines, ATP10D may play a general role in maintaining cellular GlcCer homeostasis across diverse tissues.

The capacity of cells expressing ATP10D to translocate extracellular GlcCer into the cytoplasmic leaflet and facilitate its conversion to other metabolites suggests that ATP10D could potentially contribute to the reduction of extracellular GlcCer levels, known to be an inflammatory factor (18, 19, 21, 50). Several variants of *ATP10D* are strongly associated with elevated GlcCer levels in the blood (26). Although it is uncertain whether these variants cause functional impairments in ATP10D, the *ATP10D* variants are also associated with the increased risk of insulin resistance, myocardial infarction, and atherosclerosis (25, 26, 51). Notably, the C57BL/6 mouse strain is characterized by the absence of ATP10D expression due to a premature stop codon in an *ATP10D* exon (52). This mouse strain exhibits a predisposition to develop obesity, hyperglycemia, and hyperinsulinemia when fed a high-fat diet, and these phenotypes can be alleviated through the introduction of transgenic ATP10D expression (53). Notably, these transgenic mice also demonstrated reduced levels of HexCer in both the plasma and liver tissues. Therefore, ATP10D may play a role in eliminating an excess of extracellular GlcCer. GlcCer accumulation induces an inflammatory response mediated by macrophage inducible C-type lectin (Mincle), which can serve as a GlcCer receptor, and the inflammatory response can be attenuated in Gaucher model mice lacking Mincle (20, 21, 54). Recently, it has been reported that the accumulation of GlcCer in GD can activate microglia, leading to phagocytosis of neurons and exacerbating Gaucher symptoms (50). Moreover, inhibition of GlcCer accumulation has been shown to effectively alleviate polycystic kidney disease in mouse models (55). Therefore, the clearance of ubiquitous metabolites of GlcCer is crucial for regulating immune responses and maintaining tissue integrity. The translocating activity of ATP10D toward GlcCer may be an important factor in regulating systemic GlcCer levels through clearance of GlcCer in the blood, as well as regulating intracellular GlcCer levels that contributes to maintaining lipid homeostasis.

## Data availability

Data that support the findings of this study are available from the corresponding author upon reasonable request.

## Supplemental data

This article contains supplemental data.

## Conflict of interest

The authors declare that they have no conflicts of interest with the contents of this article.
